# Metabolism of Epigenetic Ribonucleosides Leads to Nucleolar Stress and Cytotoxicity

**DOI:** 10.1021/acschembio.5c00656

**Published:** 2026-03-06

**Authors:** Xuemeng Sun, Anita Donlic, Jacob A. Boyer, Theodore E. Press, Minjae Kim, Neal K. Reddy, Clifford P. Brangwynne, Joshua D. Rabinowitz, Ralph E. Kleiner

**Affiliations:** 1 Department of Chemistry, 6740 Princeton University, Princeton, New Jersey 08544, United States; 2 Omenn-Darling Bioengineering Institute, Princeton University Princeton, New Jersey 08544, United States; 3 Department of Chemical and Biological Engineering, 6740 Princeton University, Princeton, New Jersey 08544, United States; 4 Howard Hughes Medical institute, Chevy Chase, Maryland 20815, United States; 5 Lewis-Sigler Institute for Integrative Genomics, 6740 Princeton University, Princeton, New Jersey 08544, United States; 6 Ludwig Institute for Cancer Research, 6740 Princeton University Princeton, New Jersey 08544, United States

## Abstract

Post-transcriptional RNA modifications are ubiquitous in biology, but the fate of epigenetic ribonucleotides after RNA turnover and the consequences of their metabolism and misincorporation into nucleic acids are largely unknown. Here, we explore epigenetic ribonucleoside metabolism in human cells by studying effects on cell growth, quantifying RNA misincorporation and identifying metabolic regulators, and exploring phenotypes associated with cytotoxicity. We find that bulky N^6^-modified adenosines (i.e., i^6^A) exhibit high levels of cytotoxicity and RNA misincorporation, whereas cells dramatically restrict the misincorporation of small N^6^-modified adenosines (i.e., m^6^A), partly through sanitization by enzymatic deamination, consistent with a recent report. Epigenetic ribopyrimidines also exhibit cytotoxicity, dependent on nucleoside kinase UCK2, but only at much higher concentrations than ribopurines. We further characterize the effects of cytotoxic ribonucleoside metabolism on nucleolar morphology and protein translation. Taken together, our work provides new insights into the metabolism of epigenetic ribonucleosides and mechanisms underlying their cytotoxicity to cells.

## Graphical abstract

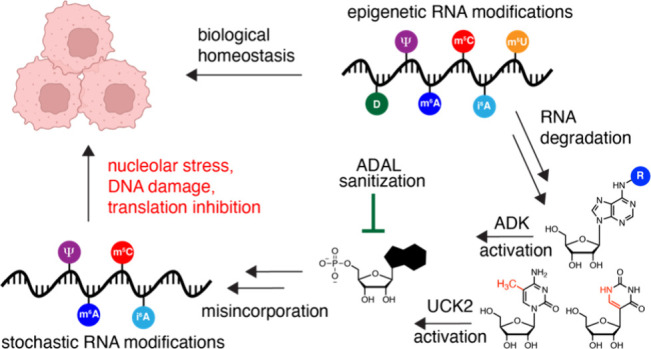

## Introduction

Nucleotides are essential for life, serving as the building blocks for RNA and DNA. Beyond their role in nucleic acid synthesis, nucleotides also participate in diverse signaling pathways. It is well established that imbalances in the nucleotide pool can disrupt cellular processes and contribute to various human diseases.^2–6^

Cells generate nucleotides through two primary biosynthetic routes: *de novo* synthesis and salvage pathways. The *de novo* pathway generates nucleotides from basic precursors including amino acids, sugars, and bicarbonate. Conversely, salvage pathways recycle nucleobases or nucleosides derived from RNA/DNA turnover or from external sources, such as dietary nucleic acids and cellular degradation products.^3,7^ These two pathways are interconnected, allowing cells to compensate for inhibition of one pathway by upregulating the other.^4,8^ Notably, the relative contributions of *de novo* synthesis and salvage vary significantly across tissues.^8^

Because the salvage pathway recycles nucleotides derived from nucleic acid turnover, this pathway is exposed not only to canonical nucleotide structures but also modified epigenetic nucleotides found in RNA/DNA. In particular, over 150 distinct chemical modifications are known on cellular RNA, spanning rRNA, tRNA, mRNA and other noncoding RNAs.^9,10^ These modifications are highly conserved across all kingdoms of life and are involved in regulating fundamental biological processes through modulation of RNA structure, base pairing, and protein-RNA interactions.^9,11,12^ Dysregulation of RNA modifications has also been implicated in numerous human diseases, including cancer and neurological disorders.^9,13,14^

Among the diverse collection of RNA modifications, those that are restricted in their abundance or exotically structured and likely incompatible with metabolism^9,13^ are unlikely to make substantial contributions to cellular nucleotide pools. In contrast, a number of modified RNA nucleotides are broadly conserved and abundant (present in concentrations ∼ 1–10% of canonical nucleotides) and structurally similar to canonical nucleotides. These include pseudouridine (Ψ),^9^ dihydrouridine (D),^9^ 5-methyluridine (m^5^U),^9^ 5-methylcytidine (m^5^C),^9^ and N^6^-methyladenosine (m^6^A),^12^ among others. Despite the prevalence of RNA modifications, the metabolic fate of modified RNA nucleotides after RNA turnover remains largely unknown. Whether specialized pathways exist to distinguish and process these structures, and what cellular consequences arise from their improper metabolism, are open questions (Figure 1A).

**Figure 1:**
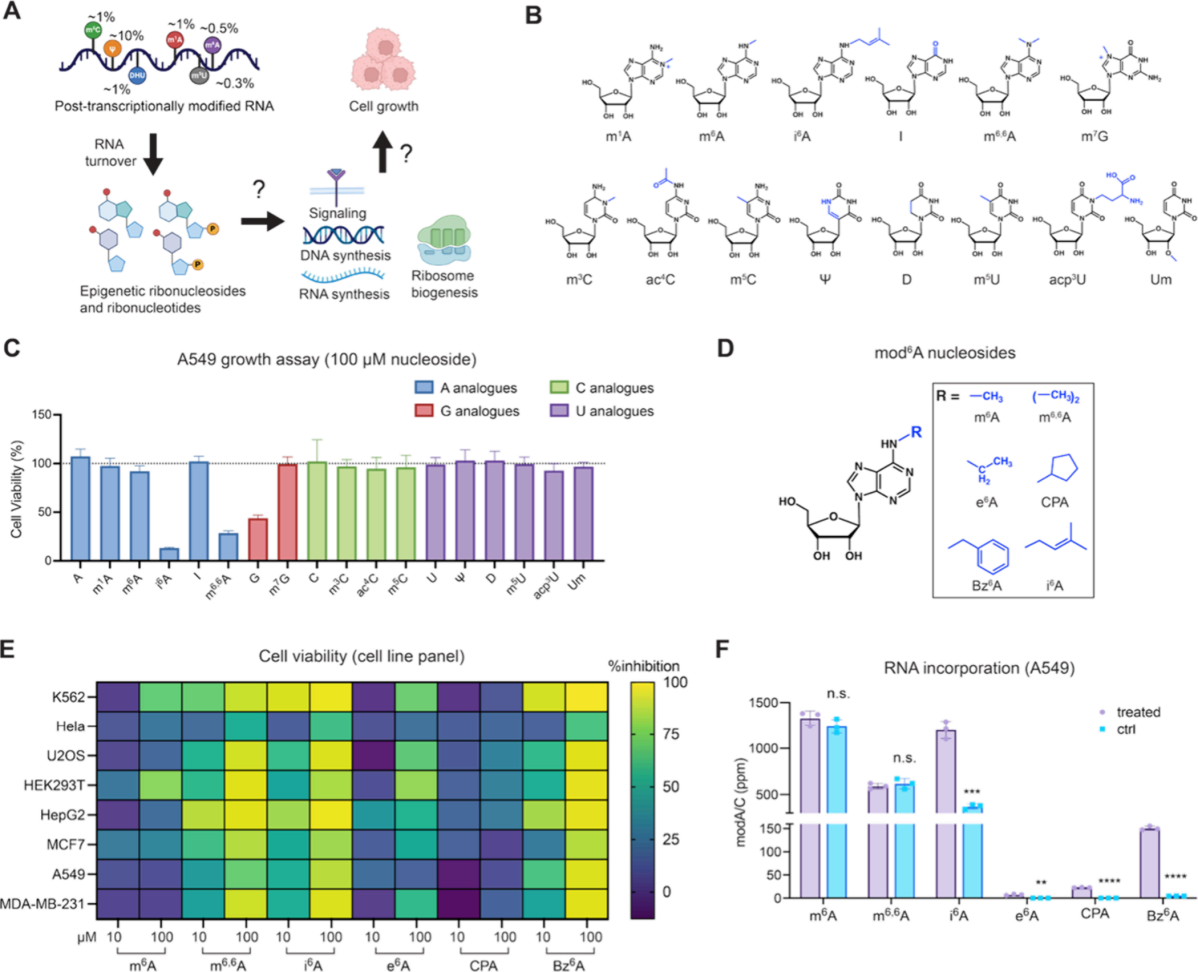
Metabolism of epigenetic ribonucleosides. (A) Scheme of potential fates of epigenetic ribonucleosides that originate from post-transcriptionally modified RNA. (B) Structures of modified epigenetic purines and pyrimidines used in this work. (C) Cytotoxicity of epigenetic ribonucleosides (100 μM for 72 h) in WT A549 cells. Cell viability was measured by an MTS-based assay and plot represents mean normalized cell viability ± s. d. (*n* = 9; three independent biological replicates with three technical replicates for each). (D) Structures of modified N^6^-adenosine analogues used in this work. (E) Screening of N^6^-modified adenosine cytotoxicity in a panel of human cell lines. Heatmap shows percentage growth inhibition with 10 μM or 100 μM nucleoside treatment for 72 h, corresponding to Supplementary Figure 1C–J. Cell viability was measured by an MTS-based assay and represents mean normalized by untreated cells. (*n* = 9 with three technical replicates for each of the three independent biological replicates). (F) RNA modification levels in total RNA after treatment with N^6^-modified adenosine analogues. WT A549 cells were treated with 10 μM nucleoside for 24 h. Modified nucleotide levels were quantified by nucleoside LC-QQQ-MS. Data are mean ± s.d. (*n* = 3). Multiple unpaired *t* test were performed between control and treated. Adjusted p values for e^6^A: *p* = 0.0024; for CPA: *p* = 0.000001; for: i^6^A: *p* = 0.00052; for Bz^6^A: *p* = 0.000002.

Previous studies investigating the metabolism of epigenetic nucleosides have focused primarily on deoxyribonucleosides. Salvage and misincorporation of the major eukaryotic epigenetic DNA modifications, 5-methylcytosine and its oxidized derivatives, are controlled by multiple metabolic enzymes in the pyrimidine salvage pathway. Direct salvage of 5-methyl-2’-deoxycytidine, the most abundant mark, is restricted by cytidine/uridine monophosphate kinase (CMPK1) and cytidine deaminase (CDA).^15^ CMPK1 also restricts salvage of 5-hydroxymethyl-2’-deoxycytidine (5hmdC) and higher oxidation states (i.e., formyl, carboxy);^15^ however, deamination to 5-hydroxymethyl-2’-deoxyuridine (5hmdU) in cells with high CDA activity results in DNA misincorporation, DNA damage, and cytotoxicity. Cells additionally express 2’-deoxynucleoside 5′-monophosphate N-glycosidase (DNPH1), a sanitization enzyme that processes 5hmdU monophosphate (5hmdUMP) and prevents its DNA misincorporation.^16^

An analogous sanitization pathway has been proposed for the abundant epigenetic ribonucleoside N^6^-methyladenosine (m^6^A). Studies in *Arabidopsis thaliana* and human cells^17^ identified N^6^-methyl-AMP deaminase (ADAL) as an enzyme that deaminates N^6^-methyl-AMP (m^6^AMP) and N^6^-methyl-2’-deoxyAMP (m^6^dAMP)^18^ into their corresponding inosine derivatives. These findings suggest that ADAL prevents metabolism and misincorporation of N^6^-methyladenosine into DNA^18,19^ and RNA; however, direct evidence for a role in preventing RNA misincorporation is lacking.^17^ Additional studies of epigenetic ribonucleosides have found that extracellular N^6^-isopentenyladenosine (i^6^A) is cytotoxic to mammalian cells and can be incorporated into cellular RNA.^20^ While our manuscript was in review, Ogawa et al. reported that the epigenetic N^6^-modified adenosine analogs m^6^A, *N* ^6^, *N* ^6^-dimethyladenosine (m^6^^,^^6^A), and i^6^A are sequentially phosphorylated by ADK and deaminated by ADAL to mitigate their cytotoxicity, linking modified adenosine metabolism to cellular stress and disease phenotypes.^1^ Beyond N^6^-modified adenosines, in *Arabidopsis*, loss of nucleoside hydrolase 1 (NSH1) correlates with accumulation of 5-methyluridine (m^5^U) into mRNA and reduced seedling growth,^21^ however mammalian NSH1 homologues have yet to be identified. Overall, the metabolic pathways mediating the activation and restriction of diverse epigenetic ribonucleosides and the consequences of RNA misincorporation of these structures are poorly annotated and understood.

In this study, we investigated a panel of epigenetic pyrimidine and purine ribonucleosides in cultured human cells. We assessed nucleoside cytotoxicity and RNA misincorporation using cell growth assays and RNA nucleoside mass spectrometry. Furthermore, by employing CRISPR-Cas9 knockouts, we examined the roles of nucleotide salvage enzymes in epigenetic ribonucleoside metabolism. Finally, we evaluated the effects of exogenous ribonucleoside treatment on nucleolar structure and protein translation. We found that purine analogs were generally more toxic than pyrimidine ribonucleosides and characterized the role of ADAL in the restriction of diverse N^6^-modified adenosine incorporation and cytotoxicity and the role of uridine cytidine kinase 2 (UCK2) in the metabolic activation of pyrimidines. Together, our work provides new insights into the metabolic fate and cellular impact of epigenetic ribonucleosides.

## Results

### Screening Cytotoxicity of Epigenetic Ribonucleosides

To investigate whether epigenetic ribonucleosides can be metabolized and perturb normal biological processes in mammalian cells, we assembled a panel of 18 biologically abundant modified and unmodified ribonucleosides, comprising 8 purines and 10 pyrimidines (Figure 1B). We screened each nucleoside for cytotoxicity in A549 cells, a human nonsmall cell lung cancer (NSCLC) line, by adding compounds into normal growth medium at 100 μM concentration and evaluating cell proliferation after 72 h. Among the canonical ribonucleosides (A, G, C, and U), only G significantly inhibited cell proliferation, reducing cell growth by 56.2% ± 3.24% at 100 μM (Figure 1C), consistent with a previous report.^6^ None of the epigenetic ribopyrimidines tested showed measurable cytotoxicity at 100 μM. Among the modified ribopurines, N^7^-methylguanosine (m^7^G), N^1^-methyladenosine (m^1^A), and inosine (I) were nontoxic. In contrast, bulky N^6^-modified adenosines such as N^6^-isopentenyladenosine (i^6^A) and N^6^, N^6^-dimethyladenosine (m^6,6^A), exhibited strong cytotoxic effects (Figure 1C). We further evaluated the cytotoxicity of i^6^A and m^6,6^A in A549 cells and found that these compounds exhibited cytotoxicity between 1 and 10 μM concentration (Supplementary Figure 1A and 1B), aligning with previous reports in diverse human cell lines.^20,22,23^ Interestingly, treatment with the structurally related ribonucleoside N^6^-methyladenosine (m^6^A) did not significantly affect the viability of A549 cells under the conditions tested (Figure 1C).

### Bulky N^6^-Modified Adenosines Exhibit Cytotoxicity and RNA Incorporation

Given the differences observed among various N^6^-modified adenosines, we expanded our panel to include synthetic adenosine analogues with diverse N^6^-substituents (Figure 1D): N^6^-ethyladenosine (e^6^A),^24^ N^6^-cyclopentyladenosine (CPA),^23^ and N^6^-benzyladenosine (Bz^6^A).^23^ We then tested these analogues together with epigenetic N^6^-modified adenosines at 10 μM and 100 μM concentration for cytotoxicity across multiple (primarily cancer-derived) cell lines, including K562, HeLa, U2OS, HEK293T, HepG2, MCF7, A549 and MDA-MB-231. A consistent pattern emerged across most cell lines: m^6,6^A, i^6^A and Bz^6^A exhibited strong cytotoxicity, reducing cell viability by more than 80% at 100 μM (Figure 1E and Supplementary Figure 1C–J), whereas m^6^A, e^6^A, and CPA were generally much less toxic at the same concentration. We did observe some differences in nucleoside sensitivity across cell lines. For example, HeLa cells were significantly less sensitive to all assayed N^6^-modified adenosines (Figure 1E and Supplementary Figure 1C–J), whereas HepG2 and K562 were most sensitive to m^6,6^A, i^6^A, and Bz^6^A. In addition, m^6^A demonstrated modest toxicity in HEK293T and K562. These differences may stem from variation in metabolic flux or signaling pathways related to nucleic acid quality control or cellular stress across individual cell types.^25,26^

To investigate whether N^6^-modfied nucleosides are metabolized and incorporated into cellular RNA, we performed quantitative nucleoside LC-QQQ-MS after nucleoside feeding in A549 cells (Supplementary Figures 2 and 3). We could not detect changes in m^6,6^A or m^6^A levels after nucleoside feeding, however the high endogenous abundance of these modifications in total RNA (∼500–1000 ppm) precludes reliable measurement of low-level metabolic incorporation. In contrast, we measured clear incorporation of i^6^A into total RNA, rising by 830 ± 99.0 ppm, a ∼ 2-fold increase over endogenous levels (Figure 1F). We measured similar i^6^A incorporation in HeLa cells, although it required higher treatment concentration than in A549, in line with the reduced cytotoxicity of i^6^A in HeLa (Supplementary Figure 4A and Figure 1E). For synthetic adenosine analogues, we detected low-level RNA incorporation for e^6^A (7.3 ± 1.4 ppm) and CPA (22.7 ± 0.5 ppm), and higher RNA incorporation for Bz^6^A (150.9 ± 4.3 ppm) (Figure 1F). These results indicate that the cytotoxicity of N^6^-modified adenosine analogues is correlated with the extent of their incorporation into RNA. Interestingly, bulkier N^6^-modifications, such as those found in Bz^6^A and i^6^A, are incorporated more efficiently into RNA than N^6^-modified adenosines with small alkyl substituents. This stands in contrast with typical metabolic incorporation trends that favor metabolic activation of nucleotide precursors more similar in size to canonical structures,^27,28^ presumably due to increased substrate compatibility with nucleotide salvage enzymes and RNA polymerases.

### ADK Activates N^6^-Modified Adenosine Analogues

To understand the metabolic pathways mediating modified adenosine activation and RNA incorporation, we investigated genes in the adenosine salvage pathway. Adenosine nucleosides can be catabolized by adenosine deaminase (ADA) followed by purine nucleoside phosphorylase (PNPase), or phosphorylated by adenosine kinase (ADK) to adenosine monophosphate (AMP), which is then further metabolized to ATP, or alternatively deaminated by AMP deaminase (AMPD) to inosine monophosphate (IMP) (Figure 2A). Since ADK is the canonical enzyme responsible for phosphorylation of adenosine to AMP in mammals,^29,30^ and deamination by ADA would remove the N^6^-substituent, we hypothesized that ADK is involved in N^6^-modified adenosine toxicity and RNA misincorporation. We therefore generated a population knockout (KO) of ADK in A549 cells (Supplementary Figure 5A,B) using CRISPR/Cas9 methods. Strikingly, depletion of ADK resulted in near complete reversal of cytotoxicity induced by N^6^-modified adenosine nucleosides, including e^6^A, m^6,6^A, i^6^A, and Bz^6^A (Figure 2B). Consistent with this finding, we observed a dramatic 80.9% ± 14.7% reduction of i^6^A and a 98.9% ± 2.0% reduction of Bz^6^A in RNA incorporation in ADK KO cells compared with WT control (Figure 2C and Supplementary Figure 4B). Further, we generated a clonal ADK KO cell line in HEK293T parent cells (Supplementary Figure 5C) and also observed resistance to the cytotoxic effects of N^6^-modified adenosines, which could be reversed by re-expression of WT ADK via plasmid transfection (Supplementary Figure 5D). Together, these data demonstrate that ADK is required for RNA incorporation and associated cytotoxicity of N^6^-modified adenosine analogues, likely by mediating their direct phosphorylation to N^6^-modified AMP nucleotides. This metabolic activation likely underlies their cytotoxic effects, further supporting a direct link between nucleoside metabolism, RNA incorporation, and cytotoxicity.

**Figure 2:**
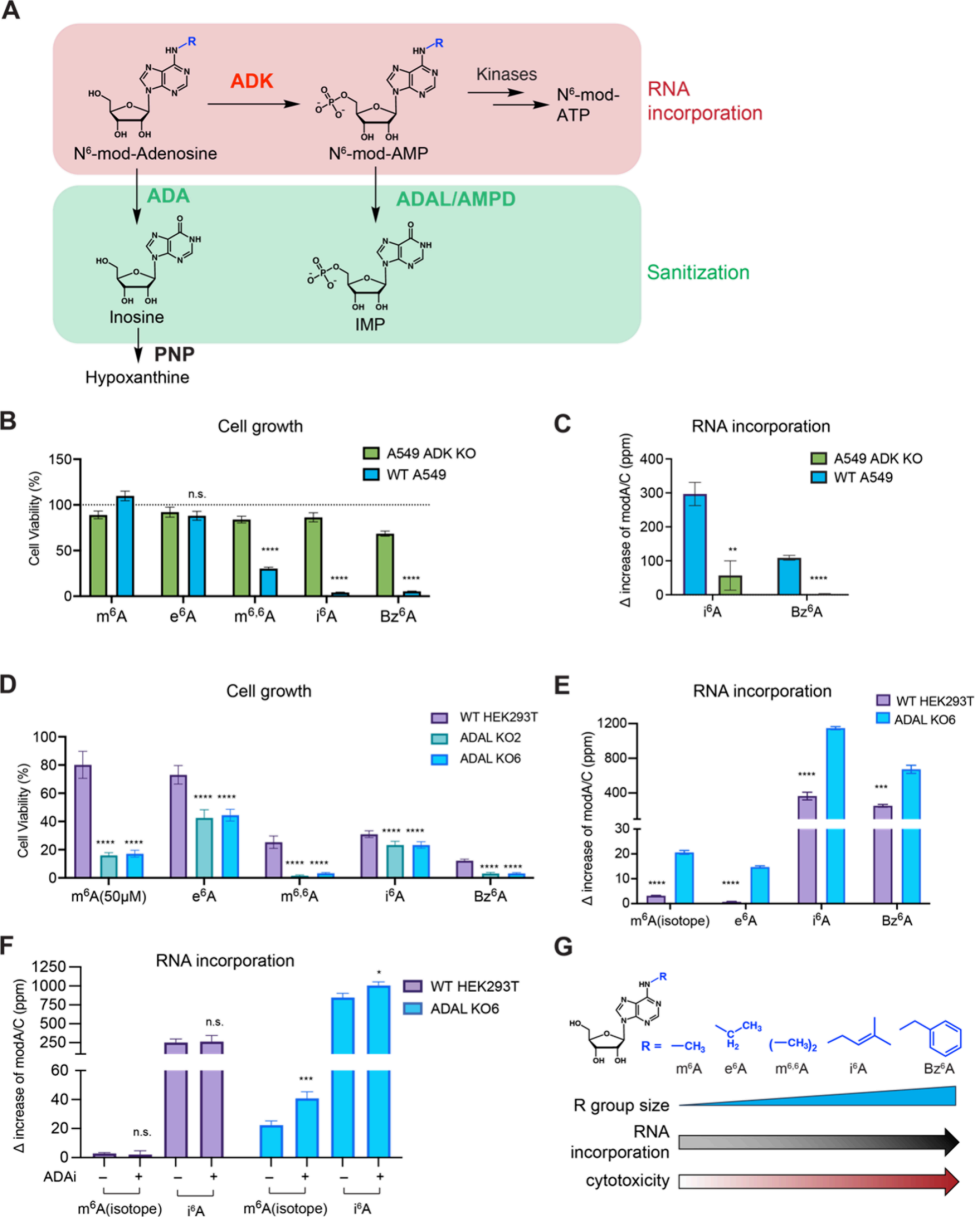
Metabolism of N^6^-modified adenosine analogues. (A) Enzymatic pathways involved in adenosine nucleoside metabolism. (B) Quantification of cell growth for A549 WT and ADK KO cells after treatment with N^6^-modified adenosines (100 μM for 72 h). Cell growth was measured using an MTS-based assay. Plot represents mean normalized cell viability ± s. d. (*n* = 9; three independent biological replicates with three technical replicates for each). Multiple unpaired *t* test were performed between WT and ADK KO. Adjusted p values for m^6^A: *p* < 0.000001; for e^6^A: *p* = 0.12; for m^6,6^A: *p* < 0.000001; for i^6^A: *p* < 0.000001; for Bz^6^A: *p* < 0.000001. (C) RNA incorporation for i^6^A and Bz^6^A in A549 WT and ADK KO cells. Cells were treated with 10 μM nucleoside for 24 h and RNA modification levels in total RNA were analyzed by nucleoside LC-QQQ-MS. Data are mean ± s.d. (*n* = 3). The unnormalized data is shown in Supplementary Figure 4B. Multiple unpaired *t* test were performed between WT and ADK KO. Adjusted p values for i^6^A: 0.001651; for Bz^6^A: 0.000014. (D) Quantification of cell growth for HEK293T WT and ADAL KO2/KO6 cells after treatment with N^6^-modified adenosine analogues (10 μM for 72 h for all analogues except m^6^A, which was performed at 50 μM for 72 h). Cell growth was measured using an MTS-based assay. Plot represents mean normalized cell viability ± s. d. (*n* = 9; three independent biological replicates with three technical replicates for each). Multiple unpaired *t* test were performed between WT and ADAL KOs. Adjusted p values for m^6^A: WT vs KO2 and WT vs KO6: *p* < 0.000001; for e^6^A: WT vs KO2 *p* < 0.000001; WT vs KO6 *p* < 0.000001; for m^6,6^A: WT vs KO2 *p* < 0.000001; WT vs KO6 *p* < 0.000001; for i^6^A: WT vs KO2 *p* = 0.000008; WT vs KO6 *p* = 0.000003; for Bz^6^A: WT vs KO2 *p* < 0.000001; WT vs KO6 *p* < 0.000001. (E) RNA incorporation for N^6^-modified adenosine analogues in HEK293T WT and ADAL KO6. Cells were treated with nucleosides (10 μM for 24 h) and modified nucleotide levels in total RNA were analyzed by nucleoside LC-QQQ-MS. Data are mean ± s.d. (*n* = 3). The unnormalized data is shown in Supplementary Figure 4C. Multiple unpaired *t* test were performed between WT and ADAL KO6. Adjusted p values for m^6^A-D_3_: *p* = 0.000008; for e^6^A: *p* = 0.000003; for i^6^A: *p* = 0.000018; for Bz^6^A: *p* = 0.000117. (F) RNA incorporation of m^6^A-D_3_ and i^6^A in HEK293T WT and ADAL KO6 with or without ADAi treatment (10 μM pentostatin). ADAi treated cells were pretreated with 10 μM pentostatin for 6 h and then exposed to both nucleosides (10 μM) and pentostatin overnight. Cells without ADAi treatment were only incubated with 10 μM nucleosides overnight and harvested together with the treated samples. Modified nucleotide levels in total RNA were quantified by nucleoside LC-QQQ-MS. Data are mean ± s.d. Three independent biological replicates were assayed for i^6^A treated cells. For m^6^A-D_3_ feeding, WT without ADAi: *n* = 6; ADAL KO6 without ADAi: *n* = 5; WT or ADAL KO6 with ADAi: *n* = 3. The unnormalized data is shown in Supplementary Figure 8D,E. Multiple unpaired *t* test were performed between ADAi treated and untreated samples. Adjusted p-values for m^6^A-D_3_: *p* = 0.00056; for i^6^A: *p* = 0.03. (G) Scheme that summarizes the trend of cytotoxicity and RNA incorporation with modification group size at N^6^ position.

### Sanitization of N^6^-Modified Adenosines by ADAL and ADA

Since the metabolic incorporation of N^6^-modified adenosines followed an unexpected structural trend (i.e., larger derivatives were incorporated more readily than smaller derivatives), we explored whether selective sanitization pathways may exist for smaller N^6^-modified adenosine nucleosides that closely resemble epigenetic nucleosides (i.e., m^6^A). An active sanitization pathway for N^6^-modified adenosines that preferentially removes smaller nucleotides from the nucleotide pool would explain the inefficient metabolism and RNA incorporation of these compounds. We focused our efforts on adenosine deaminase-like protein (ADAL) (Figure 2A), as previous studies have shown that ADAL can deaminate m^6^AMP and m^6^dAMP and prevent misincorporation of N^6^-methyladenine into DNA;^18,19^ whether ADAL prevents the misincorporation of N^6^-modified adenosines into RNA is poorly understood. We investigated the role of ADAL in N^6^-modified adenosine sanitization by generating clonal ADAL KO cell lines in HEK293T and HeLa (Supplementary Figure 6A-C, Supplementary Figure 7A,B). ADAL KO cells showed no growth defects (Supplementary Figure 6D) and no disruption in endogenous levels of m^6^A, m^6,6^A, or i^6^A (Supplementary Figure 6E). However, deletion of ADAL sensitized cells to all five N^6^-modified adenosine analogues tested, as observed in two independent HEK293T ADAL KO lines and two independent HeLa KO lines (Figure 2D and Supplementary Figure 7C). These effects could be rescued by re-expression of WT ADAL enzyme by plasmid transfection (Supplementary Figure 7D). As the effects were more pronounced in HEK293T, we further focused on these cell lines. Consistent with increased cytotoxicity, ADAL KO also led to increased RNA incorporation of all five analogues (Figure 2E, Supplementary Figure 4C,D) with 7-fold or more increase over WT for m^6^A-D_3_ (+17.5 ± 0.8 ppm) and e^6^A (+14.0 ± 0.5 ppm), and ∼ 3-fold increase over WT for i^6^A (+782 ± 47.6 ppm) and Bz^6^A (+420 ± 48.6 ppm). We also found that m^6^A-D_3_ incorporation in poly­(A) RNA was comparable to that in total RNA (primarily rRNA/tRNA) in ADAL KO cells (Supplementary Figure 4E), suggesting that it can be incorporated into RNA by different polymerases.

Consistent with previous reports showing that ADAL prevents misincorporation of ribonucleotide m^6^A into genomic DNA,^18,19^ we measured elevated levels of m^6^dA in our ADAL KO cells that were not fed exogenous m^6^A ribonucleoside (Supplementary Figure 6F). Interestingly, we were not able to measure increased m^6^dA levels in the genome of WT or ADAL KO cells upon feeding exogenous isotopically labeled m^6^A-D_3_ at 50 μM for 2 days (data not shown). We speculate that longer-term feeding or higher nucleoside concentrations would be required to see perturbation of genomic m^6^dA levels; indeed previous studies^19^ used prolonged incubation with m^6^A-D_3_ to generate detectable levels in genomic DNA.

Whereas ADAL KO sensitized cells to diverse N^6^-modified adenosines and concomitantly increased their incorporation into RNA, incorporation of small alkyl-substituted m^6^A and e^6^A into RNA still proceeded ∼ 30–70-fold less efficiently than larger i^6^A and Bz^6^A derivatives in the ADAL KO lines. We therefore explored whether sanitization pathways upstream of ADAL, such as deamination by nucleoside deaminases (i.e., ADA1, ADA2),^31^ may prevent the metabolism of small N^6^-modified nucleosides. Notably, ADA1 shares a high degree of structurally similarity with ADAL, with a root-mean-square-deviation (RMSD) of only 1.56 Å (Supplementary Figure 8A–C)). To inhibit the activity of ADA proteins, we treated WT and ADAL KO cells with pentostatin, an established and potent small molecule inhibitor of ADA1/2 enzyme,^32,33^ and measured RNA incorporation of m^6^A-D_3_ and i^6^A nucleosides. Pentostatin treatment modestly increased RNA incorporation of m^6^A-D_3_ in ADAL KO cells (82% increase), but not in WT cells, and had minimal effect on incorporation of i^6^A (19% increase in KO) (Figure 2F and Supplementary Figure 8D,E). Collectively, our results indicate that diverse N^6^-modified adenosine analogues are activated by ADK and incorporated into RNA. Bulky N^6^-modified adenosines exhibit the highest levels of cytoxicity and RNA incorporation, and cells can mitigate misincorporation and cytotoxicity of N^6^-modified adenosine analogues through sanitization pathways involving nucleotide and nucleoside deaminases, ADAL and ADA (Figure 2G); however, even in the absence of ADAL/ADA-mediated sanitization pathways, m^6^A and structurally related derivatives show limited RNA misincorporation, suggesting that other mechanisms exist to prevent stochastic RNA incorporation of epigenetic N^6^-modified adenosines.

### Cytotoxicity of Epigenetic Pyrimidine Ribonucleosides

Although epigenetic ribopyrimidines did not show cytotoxic effects in our initial screen at 100 μM concentration in A549 cells (Figure 1C), we repeated the screen at higher concentrations and expanded to additional human cell lines. In A549, we began to see cytotoxic effects at 5 mM concentration (Figure 3B and Supplementary Figure 9A). Among the compounds tested, dihydrouridine (D) inhibited cell growth by 83.7% ± 1.3%, 5-methyluridine (m^5^U) by 50.3% ± 4.0%, 5-methylcytidine (m^5^C) by over 90%, and 2’-O-methylcytidine (Cm) by 63.8% ± 2.6% (Figure 3B). These results indicate that epigenetic ribopyrimidines can be cytotoxic, but only at high (millimolar) concentrations. We then extended the assay to a panel of cell lines. At 1 mM treatment, m^5^C consistently exhibited the strongest cytotoxicity, inhibiting growth by over 90% in HeLa and HEK293T cells (Figure 3C and Supplementary Figure 9C–H). Interestingly, pseudouridine (Ψ) showed cell-type specific effects, with more than 90% inhibition in HeLa cells, but minimal toxicity in A549 and MCF7. Moderate inhibition (∼30%) was observed in HepG2, U2OS and HEK293T cells. When the treatment concentration was further increased to 5 mM, the cytotoxicity patterns become more pronounced: D, m^5^C, and m^5^U were broadly toxic across most cell lines, while Ψ remained selectively toxic in specific cell types (Supplementary Figure 9B–H).

**Figure 3:**
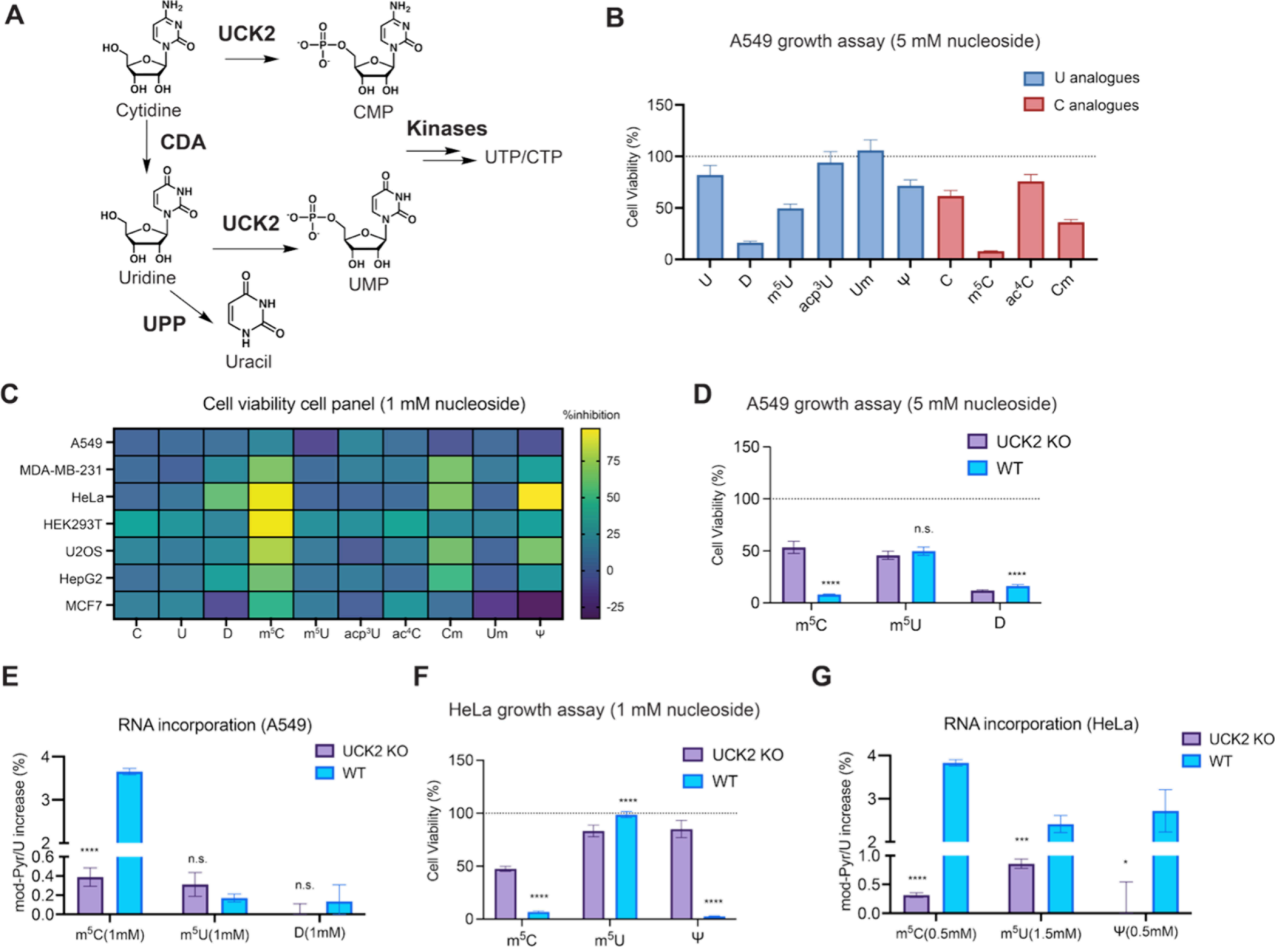
Metabolism of modified ribopyrimidine analogues. (A) Metabolic pathways involved in ribopyrimidine metabolism. (B) Cytotoxicity of epigenetic ribonucleosides (5 mM for 72 h) in WT A549 cells. Cell viability was measured by MTS assay and plot represents mean normalized cell viability ± s. d. (*n* = 9; three independent biological replicates with three technical replicates for each). (C) Ribopyrimidine nucleoside cytotoxicity in a panel of human cell lines. Heatmap shows percentage growth inhibition (1 mM nucleoside for 72 h), corresponding to Supplementary Figure 9A and C–H. Cell viability was measured by an MTS-based assay and represents mean normalized by untreated cells. (*n* = 9 with three technical replicates for each of the three independent biological replicates). (D) Quantification of cell growth for A549 WT and UCK2 KO cells with m^5^C, m^5^U or D treatment. Cell growth was measured by MTS assay after nucleoside treatment (5 mM for 72 h). Plot represents mean normalized cell viability ± s. d. (*n* = 9; three independent biological replicates with three technical replicates for each). Multiple unpaired *t* test were performed between WT and UCK2 KO. Adjusted p values for m^5^C: *p* < 0.000001; for D: *p* < 0.000001. (E) RNA incorporation levels for m^5^C, m^5^U, or D. A549 WT or UCK2 KO cells were treated with 1 mM nucleoside for 24 h and RNA modification levels in total RNA were quantified by nucleoside LC-QQQ-MS. Data are mean ± s.d. (*n* = 3). The unnormalized data is shown in Supplementary Figure 10E. Multiple unpaired *t* test were performed between WT and UCK2 KO. Adjusted p value for m^5^C: *p* = 0.000004. (F) Quantification of cell growth for HeLa WT and UCK2 KO cells after treatment with m^5^C, m^5^U or Ψ (1 mM for 72 h). Cell growth was measured by MTS assay. Plot represents mean normalized cell viability ± s. d. (*n* = 9; three independent biological replicates with three technical replicates for each). Multiple unpaired *t* test were performed between WT and UCK2 KO. Adjusted p values for m^5^C: *p* < 0.000001; for m^5^U: *p* = 0.000009; for Ψ *p* < 0.000001. (G) RNA incorporation levels for m^5^C, m^5^U or Ψ in HeLa WT or UCK2 KO cells. Modified nucleotide levels were quantified in total RNA by nucleoside LC-QQQ-MS after m^5^C/ Ψ (0.5 mM) or m^5^U (1.5 mM) treatment for 24 h. Data are mean ± s.d. (*n* = 3). Unnormalized data is shown in Supplementary Figure 11D. Multiple unpaired *t* test were performed between WT and UCK2 KO. Adjusted p values for m^5^C: *p* < 0.000001; for m^5^U: *p* = 0.00045; for Ψ: *p* = 0.018.

### UCK2 Mediates the Incorporation and Toxicity of Epigenetic Ribopyrimidines

Our group^34–36^ and others^37–40^ have previously established that uridine-cytidine kinase 2 (UCK2), which phosphorylates uridine/cytidine nucleosides to NMPs, is rate-limiting for uptake and RNA incorporation of modified pyrimidine ribonucleosides (Figure 3A). To explore its role in metabolism of epigenetic ribopyrimidines, we generated UCK2 KO cells in A549 (Supplementary Figure 10A) and HeLa (Supplementary Figure 11A) and assayed sensitivity to D, m^5^C and m^5^U. In A549, UCK2 depletion markedly rescued the cytotoxicity induced by m^5^C, while no significant rescue was observed for D or m^5^U treatments (Figure 3D and Supplementary Figure 10B,C). Through dose–response titrations, we observed >7-fold increase in the IC_50_ of m^5^C in A549 cells with UCK2 depletion. In contrast, the IC_50_ values for m^5^U and D remained nearly unchanged between UCK2 KO and WT cells (Supplementary Figure 10D). Using nucleoside LC-MS/MS, we quantified RNA incorporation (Supplementary Figure 12) and detected 3.65% ± 0.07% incorporation of m^5^C in WT A549 cells (a 4-fold increase over endogenous m^5^C levels), compared to only 0.38% ± 0.096% incorporation in UCK2 KO cells (Figure 3E and Supplementary Figure 10E). In contrast, D and m^5^U levels did not significantly change between UCK2 KO and WT (Figure 3E and Supplementary Figure 10E), consistent with similar cytotoxicity profiles (Figure 3D).

Similar trends were observed in HeLa cells. UCK2 KO rescued m^5^C-induced toxicity but not m^5^U toxicity (Figure 3F and Supplementary Figure 11B), suggesting m^5^U is a poor substrate for UCK2. As noted above, HeLa cells were selectively sensitive to Ψ, an effect that we found to be dependent on UCK2 status (Figure 3F and Supplementary Figure 11B,C). This effect was less pronounced but still evident in A549 cells (Supplementary Figure 10C). IC_50_ values for m^5^C and Ψ in HeLa UCK2 KO cells increased ∼4–5-fold as compared to WT cells (Supplementary Figure 11C). In WT HeLa cells, LC-QQQ-MS quantification revealed UCK2-dependent incorporation of m^5^C at 3.83% ± 0.07% and Ψ at 2.72% ± 0.49% following 0.5 mM treatment in WT HeLa (Figure 3G and Supplementary Figure 11D). Surprisingly, although m^5^U did not show UCK2-dependent toxicity in HeLa, we detected UCK2-dependent RNA incorporation: m^5^U levels increased by ∼ 14.5-fold in WT (2.41% ± 0.20% incorporation) and by only 5-fold in UCK2 KO (0.86% ± 0.08% incorporation) (Figure 3G and Supplementary Figure 11D). While UCK2 depletion did not rescue m^5^U toxicity, the underlying mechanism remains unclear. It is possible that metabolism-independent processes or alternative UCK2-independent metabolic pathways contribute to m^5^U cytotoxicity. Further, we demonstrated rescue of pyrimidine cytotoxicity in UCK2 KO cells by re-expression of WT UCK2 by plasmid transfection (Supplementary Figure 11E). Taken together, these results demonstrate that UCK2 is the key enzyme responsible for m^5^C and Ψ toxicity via its role in promoting their RNA incorporation. In contrast, m^5^U and D show minimal UCK2 dependence, suggesting distinct metabolism or cytotoxicity stemming from the unprocessed nucleosides.

### Pyrimidine Ribonucleoside Cytotoxicity Induces DNA Damage but Is Not Associated with DNA Incorporation

We next investigated the mechanisms by which modified ribonucleosides cause cell death. Given the high concentrations of epigenetic pyrimidine ribonucleosides needed for cytotoxicity, and associated high levels of RNA incorporation, we questioned whether modified pyrimidines fed in this way may also be metabolized to modified deoxynucleotides and possibly incorporated into DNA, thereby inducing DNA damage or replication stress/inhibition. First, we investigated whether modified ribonucleoside treatment induced DNA damage in A549 or HeLa cells. In HeLa cells, treatment with m^5^C, Ψ, or m^5^U for 48h at concentrations that induced comparable RNA incorporation (∼3%) resulted in DNA damage as measured by γH2AX Western blot, whereas i^6^A treatment did not increase γH2AX signal in either cell line (Supplementary Figure 13A,B). In A549, γH2AX induction was more modest; however, m^5^C treatment did result in a slight increase in DNA damage-associated signal. We next focused on quantifying DNA incorporation and deoxynucleotide metabolites derived from m^5^C because it was the most toxic epigenetic ribopyrimidine we assayed, it induced DNA damage in HeLa and A549 cells, and corresponding modified deoxyribonucleoside standards for LC-MS analysis were available. We extracted genomic DNA from treated and untreated cells and analyzed 5-methyl-2’-deoxycytidine (5mdC) levels by nucleoside LC-QQQ-MS. Upon m^5^C feeding, we did not observe significant differences in 5mdC levels in either A549 or HeLa cells (Supplementary Figure 13C,D). However, considering the high endogenous abundance of 5mdC in genomic DNA, it is possible that subtle changes might be masked in bulk LC-QQQ-MS analysis. Further, we examined the nucleotide pools and found no detectable formation of m^5^dCTP after 2 or 24 h m^5^C treatments (Supplementary Figure 14). Instead, we observed a marked accumulation of m^5^C monophosphate (m^5^CMP) and m^5^C triphosphate (m^5^CTP), as well as the corresponding deamination products, m^5^U monophosphate (m^5^UMP) and m^5^U triphosphate (m^5^UTP) (Supplementary Figure 14), aligning with increases in m^5^C in RNA (Figure 3G and Supplementary Figure 11D). The abundance of m^5^C and m^5^U-derived nucleotide was higher 2 h postfeeding than 24 h, indicating that cells were actively metabolizing these molecules. In contrast, whereas m^5^C nucleoside levels also declined from 2 to 24 h, m^5^U nucleoside and 5-methyluracil (thymine) accumulated in the cell from 2 to 24 h. Taken together, our findings show that epigenetic ribopyrimidine-induced cytotoxicity correlates with DNA damage but appears to be independent of metabolism to modified deoxynucleotides or DNA incorporation.

### i^6^A and Ψ Induce Nucleolar Stress through Distinct Mechanisms

Active rDNA transcription is essential for nucleolar organization, and nucleolar function is tightly linked to its structure as a multiphase condensate.^41–44^ The mammalian nucleolus consists of three subcompartments: the fibrillar center (FC), dense fibrillar component (DFC) and granular component (GC). rDNA is transcribed at the FC-DFC interface, pre-rRNA processing occurs in the DFC, and ribosomal subunit assembly takes place in the GC.^41–45^ Inhibition of rDNA transcription triggers nucleolar stress, characterized by redistribution of nucleolar proteins to the periphery, leading to a rounded, condensed appearance.^42–45^ This can be visualized using markers specific to different nucleolar compartments.^43,45^

To examine whether epigenetic ribonucleoside cytotoxicity and RNA incorporation correlate with perturbation of nucleolar structure and function, which could lead to defects in ribosome biogenesis and protein translation and arrest cell cycle progression, we examined nucleolar morphology by staining for nucleophosmin (NPM1), a GC marker, and RPA-194 (RNA Polymerase I Subunit A), an FC marker. In HeLa cells treated with i^6^A treatment, we observed nucleolar caps forming around the GC (Figure 4A), a hallmark of nucleolar segregation caused by Pol I inhibition.^46,47^ Quantification of nucleolar eccentricity showed a 27% decrease (Figure 4B), reflecting nucleolar rounding in the images (Figure 4A) and consistent with Pol I inhibition.^48,49^ In addition, NPM1 partitioning was significantly altered by i^6^A treatment (Figure 4C). While Ψ treatment did not induce nucleolar caps, it did cause nucleolar rounding, as evidenced by a 14% decrease in eccentricity (Figure 4A,B). These morphological changes suggest that Ψ also induces nucleolar stress, albeit likely through a mechanism distinct from Pol I inhibition. In contrast, m^5^C treatment had no obvious effect on nucleolar morphology (Figure 4A,B).

**Figure 4:**
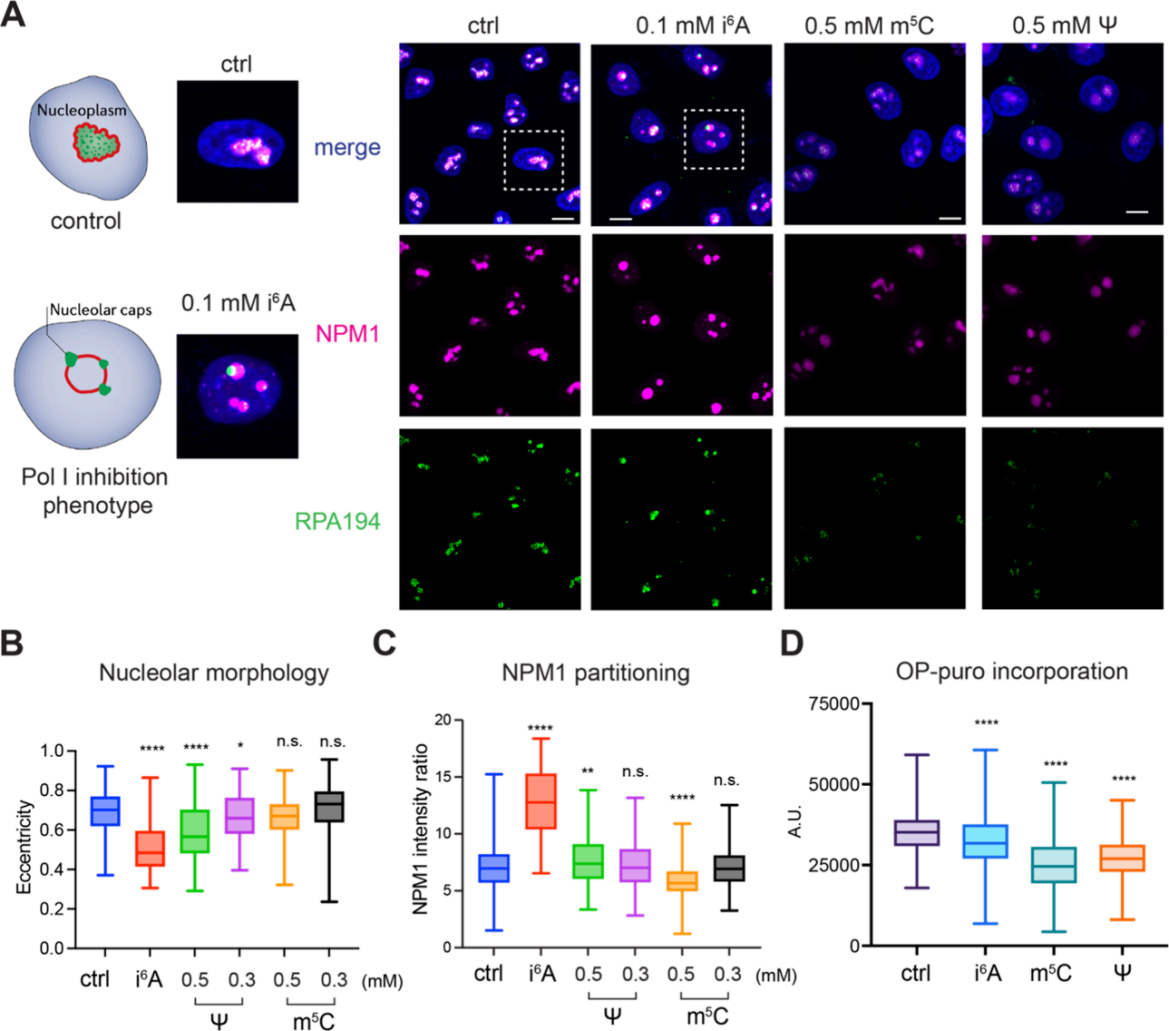
Epigenetic nucleosides induce nucleolar stress and global translation inhibition. (A) Confocal micrographs of HeLa cells treated with 100 μM i^6^A, 500 μM m^5^C or 500 μM Ψ for 24h. Cells were stained with antibodies for NPM1 and RPA-194 and a nuclear dye. Scale bar represents 10 μm. Representative images from two biological replicates are shown. (B) Quantification of nucleolus morphology (eccentricity). Boxes in the plot represent 25th to 75th percentiles and whiskers show min to max. Two tailed unpaired *t* test was performed between treated and control samples and p values for i^6^A: *p* < 0.000001; Ψ (0.5 mM): *p* = 0.000011; Ψ (0.3 mM): *p* = 0.047; 40–120 cells were analyzed per condition. (C) Quantification of NPM1 partitioning. Boxes in the plot represent 25th to 75th percentiles and whiskers show min to max. Two tailed unpaired *t* test was performed between treated and control samples and p values for i^6^A: *p* < 0.000001; Ψ (0.5 mM): *p* = 0.0062; m^5^C: *p* < 0.000001. Between 40 and 300 cells were analyzed per condition. (D) Global translation levels upon nucleoside feeding. Quantification of OP-puro incorporation (Cy3 signal) from HeLa cells treated with 100 μM i^6^A, 500 μM m^5^C, or 500 μM Ψ for 24h. Boxes in the plot represent 25th to 75th percentiles and whiskers show min to max. Two tailed unpaired *t* test were performed between treated and control samples and p values are for i^6^A: *p* < 0.000001; for m^5^C: *p* < 0.000001; for Ψ: *p* < 0.000001.

We observed similar nucleolar perturbation with i^6^A treatment in A549 cells (Supplementary Figure 15A), though changes in eccentricity and NPM1 partitioning were less pronounced (Supplementary Figure 15B,C). These differences likely reflect cell-type-specific responses. Nonetheless, the consistent formation of nucleolar caps upon i^6^A treatment in both HeLa and A549 cells supports its role in Pol I inhibition. In contrast, m^5^C treatment did not induce noticeable alterations in A549 cells (Supplementary Figure 15), consistent with observations in HeLa cells (Figure 4A). Together, these findings suggest that i^6^A inhibits cell growth by inhibiting Pol I and inducing nucleolar stress, presumably by first misincorporating into rRNA. Ψ also induces nucleolar stress, possibly by interfering with ribosome biogenesis at the level of pre-rRNA processing or modification, given the role of pseudouridylation in these processes.^50^ The absence of these effects with m^5^C suggests its cytotoxicity operates through mechanisms unrelated to nucleolar function.

### Epigenetic Nucleosides Inhibit Global Translation

Finally, we employed *O*-propargyl-puromycin (OP-puro),^51^ which incorporates into nascent polypeptides, to assess global protein synthesis after epigenetic nucleoside treatment. We observed a marked reduction in OP-puro signal following epigenetic nucleoside treatments: 27.7% ± 1.4% (mean ± SEM) decrease for m^5^C, 22.9% ± 1.2% (mean ± SEM) decrease for Ψ, and 8.6% ± 1.4% (mean ± SEM) decrease for i^6^A (Figure 4D and Supplementary Figure 16). These results indicated that all three epigenetic nucleosides disrupt global translation to varying extents.

## Discussion

In this study, we show that a subset of epigenetic ribonucleoside can be metabolized by cells and incorporated into RNA, with associated cytotoxicity. We found that N^6^-modified adenosines were the most toxic of those tested and further showed that their metabolic activation relies upon ADK and is opposed by ADAL- and ADA-mediated sanitization pathways. Using a broader panel of epigenetic and synthetic N^6^-modified adenosines, we found a strong restriction against the misincorporation of adenosine analogues with small N^6^-alkyl substitutions such as the abundant epigenetic ribonucleoside m^6^A. Compared to purine analogues, pyrimidines were less toxic but demonstrated measurable UCK2-dependent and UCK2-independent cytotoxicity at high concentrations in multiple cell lines. Furthermore, we show that i^6^A and Ψ misincorporation correlates with nucleolar stress and global translation inhibition.

Our findings on the role of ADAL in deaminating N^6^-methyladenosine nucleotides align with previous work in *Arabidopsis* and human cells, where ADAL enzymes were shown to serve a similar protective function.^17^ Additionally, we show that ADAL has broad substrate specificity and can process native and synthetic N^6^-modified adenosines including i^6^A, Bz^6^A, and e^6^A, and prevent their misincorporation into RNA. Similarly, ADK possesses broad substrate specificity and can activate diverse functionalized N^6^-modified adenosines. While this manuscript was in preparation, similar findings were reported by Ogawa et al.^1^ By measuring RNA misincorporation rates of different N^6^-modified adenosines, we found that larger N^6^-substituents, such as isopentenyl and benzyl, exhibited incorporation at >100-fold higher levels than small alkyl N^6^-substitutions (i.e., methyl and ethyl) (Figure 2E). This is in stark contrast to typical metabolic incorporation trends for modified nucleosides that favor activation and incorporation of nucleosides that more closely resemble canonical nucleoside structures. While the precise mechanism underlying the preference for misincorporation of larger N^6^-modified adenosines remains to be determined, we speculate that it likely results from a combination of factors including the ability of larger analogues to evade catabolic adenosine pathways as well as additional layers of selectivity which may include nucleoside transporters, RNA polymerases, and sanitization pathways functioning downstream of RNA synthesis. One possible explanation is that ADAL more efficiently deaminates small N^6^-modified adenosines, as was suggested by an *in vitro* report.^52^ However, even in ADAL KO cells larger N^6^-modified adenosines still incorporate into RNA at >100-fold higher levels than m^6^A or e^6^A. This suggests that ADAL is not the only (and perhaps not even the major) restriction mechanism for preventing m^6^A misincorporation. We show that adenosine deaminase (ADA) also serves a role (albeit a minor one) in sanitizing exogenous m^6^A nucleosides. Additional studies will be required to disentangle the relative contributions of these enzymes and pathways to modified adenosine metabolism and RNA misincorporation.

In contrast to purine ribonucleosides, pyrimidines (both modified and unmodified) did not exhibit cytotoxicity except at millimolar concentrations. At these high concentrations, cytotoxicity of epigenetic pyrimidine ribonucleosides largely depended upon UCK2, which aligns with previous studies implicating UCK2 as the rate-limiting enzyme for salvage of cytotoxic pyrimidine ribonucleoside analogues.^34–40^ UCK2 is known to have stringent substrate selectivity^35,53^ and therefore this kinase (and NMP kinase CMPK1) may serve as a filter to prevent metabolism and subsequent RNA misincorporation of epigenetic pyrimidines. Accordingly, pyrimidine toxicity may only emerge when this filter capacity is exceeded at supraphysiological concentrations, allowing sufficient nucleotide accumulation and RNA misincorporation to perturb RNA function. Whereas we measured high levels of RNA misincorporation correlating with cytotoxicity, it is unknown whether cells tolerate lower concentrations of these nucleosides due to inefficient metabolism (or efficient sanitization) or lack of major perturbation to RNA structure/function upon misincorporation of pyrimidine modifications. Catabolic enzymes for Ψ (the most abundant RNA modification) have been reported in bacteria^54^ and in plants,^55^ but homologous pathways in mammals are not known.

We evaluated the cytotoxicity mechanisms of epigenetic ribonucleosides by characterizing nucleolar morphology and measuring protein translation. Several synthetic nucleosides and nucleobases are known to induce nucleolar stress, including 5-fluorouracil,^56^ 5-fluorouridine,^56^ and 4-thiouridine.^57^ Additionally, DHODH inhibitors have been shown to disrupt nucleotide balance and trigger nucleolar rounding.^58^ In our studies, modified epigenetic ribonucleosides can similarly perturb nucleolar function but through diverse mechanisms. Treatment with i^6^A induces nucleolar caps, consistent with Pol I inhibition. Yakita et al. also demonstrated that i^6^A inhibits Pol I transcription by RT-qPCR analysis of rRNA transcripts.^20^ Since i^6^A misincorporates into RNA, we speculate that it may serve as a transcription terminator. In contrast, Ψ induces nucleolar stress without significant nucleolar cap formation, suggesting that Ψ misincorporation may perturb later steps of rRNA processing and ribosome biogenesis,^50^ rather than inhibiting Pol I transcription. Consistent with this idea, studies in *E. coli* have shown that excessive Ψ in rRNA can impair ribosome assembly.^59^ Future experiments measuring the accumulation of pre-rRNA intermediates will be required to directly test this mechanism in mammalian cells. Whereas m^5^C did not induce apparent nucleolar stress based on FC and GC morphology, it did inhibit protein translation (as did i^6^A and Ψ). Therefore, m^5^C may act at later stages of ribosome biogenesis, or translation inhibition may be due to mRNA misincorporation; indeed, m^5^C modifications in synthetic mRNA are known to perturb translation efficiency.^60^ It is important to note that whereas our study demonstrates a clear correlation between RNA misincorporation and cytotoxicity, we also cannot exclude the possibility that a modified nucleotide metabolite (rather than improperly modified RNA) is the cytotoxic species in cells. Pinning down this connection will require additional mechanistic studies in the future.

In summary, our study provides new insights into the cellular metabolism of epigenetic ribonucleosides and highlights the role of nucleotide salvage and sanitization pathways in managing their incorporation into RNA. These findings deepen our understanding of the metabolic fate of RNA modifications following RNA turnover and offer valuable insights into the cellular handling of nucleoside-based drugs with similar structures.

## Supplementary Material



## Notes

Experimental materials and methods, nucleoside and nucleotide LC-MS data, KO cell line validation, cell growth assays, DNA damage assays, protein translation assays, nucleolar microscopy (PDF)

## References

[1] Ogawa A., Watanabe S., Ozerova I., Tsai A. Y. L., Kuchitsu Y., Chong H. B., Kawakami T., Fuse J., Han W., Kudo R., Naito T., Sato K., Nakazawa T., Saheki Y., Hirayama A., Stadler P. F., Arisawa M., Araki K., Bar-Peled L., Taguchi T., Sawa S., Inaba K., Wei F. Y. (2025). Adenosine kinase and
ADAL coordinate detoxification of modified adenosines to safeguard
metabolism. Cell.

[2] Aird K. M., Zhang R. (2015). Nucleotide metabolism, oncogene-induced
senescence and cancer. Cancer Lett..

[3] Lane A. N., Fan T. W.-M. (2015). Regulation of
mammalian nucleotide metabolism and biosynthesis. Nucleic Acids Res..

[4] Mullen N. J., Singh P. K. (2023). Nucleotide metabolism: a pan-cancer metabolic dependency. Nat. Rev. Cancer.

[5] Sprenger H.-G. (2021). Cellular pyrimidine imbalance triggers mitochondrial
DNA–dependent
innate immunity. Nat. Metab..

[6] Diehl F. F. (2022). Nucleotide imbalance
decouples cell growth from cell proliferation. Nat. Cell Biol..

[7] Ali E. S., Ben-Sahra I. (2023). Regulation of nucleotide metabolism
in cancers and
immune disorders. Trends Cell Biol..

[8] Tran D. H. (2024). De novo and salvage
purine synthesis pathways across tissues and
tumors. Cell.

[9] McCown P. J. (2020). Naturally occurring modified ribonucleosides. WIREs RNA.

[10] Cappannini A. (2024). MODOMICS: a database
of RNA modifications and related information.
2023 update. Nucleic Acids Res..

[11] Ontiveros R. J., Stoute J., Liu K. F. (2019). The chemical
diversity of RNA modifications. Biochem. J..

[12] Seo K. W., Kleiner R. E. (1975). Mechanisms of epitranscriptomic
gene regulation. Biopolymers.

[13] Suzuki T. (2021). The expanding
world of tRNA modifications and their disease relevance. Nat. Rev. Mol. Cell Biol..

[14] Jonkhout N. (2017). The RNA modification
landscape in human disease. RNA.

[15] Zauri M. (2015). CDA directs metabolism
of epigenetic nucleosides revealing a therapeutic
window in cancer. Nature.

[16] Fugger K. (2021). Targeting the nucleotide
salvage factor DNPH1 sensitizes *BRCA* -deficient cells
to PARP inhibitors. Science.

[17] Chen M. (2018). m ^6^ A RNA
Degradation Products Are Catabolized by an Evolutionarily
Conserved N ^6^ -Methyl-AMP Deaminase in Plant and Mammalian
Cells. Plant Cell.

[18] Chen S., Lai W., Li Y., Liu Y., Jiang J., Li X., Jiang G., Wang H. (2023). Aberrant DNA N ^6^ -methyladenine incorporation via adenylate
kinase 1 is suppressed
by ADAL deaminase-dependent 2′-deoxynucleotide pool sanitation. EMBO J..

[19] Musheev M. U., Baumgärtner A., Krebs L., Niehrs C. (2020). The origin of genomic
N6-methyl-deoxyadenosine in mammalian cells. Nat. Chem. Biol..

[20] Yakita M. (2022). Extracellular *N*
^
*6*
^ -isopentenyladenosine
(i ^6^ A) addition induces cotranscriptional i ^6^ A incorporation into ribosomal RNAs. RNA.

[21] Gao S., Sun Y., Chen X., Zhu C., Liu X., Wang W., Gan L., Lu Y., Schaarschmidt F., Herde M., Witte C. P., Chen M. (2023). Pyrimidine catabolism is required to prevent the accumulation
of 5-methyluridine in RNA. Nucleic Acids Res..

[22] Slocum H. K., Hakala M. T. (1975). Mechanism of Natural
Resistance to N6-(&-Isopentenyl)­adenosine
in Cultured Cells’. Cancer Res..

[23] Ottria R., Casati S., Baldoli E., Maier J. A. M., Ciuffreda P. (2010). N6-Alkyladenosines:
Synthesis and evaluation of in vitro anticancer activity. Bioorg. Med. Chem..

[24] Dunwiddie T. V., Worth T. S., Olsson R. A. (1986). Adenosine analogs
mediating depressant
effects on synaptic transmission in rat hippocampus: Structure-activity
relationships for the N6 subregion. Naunyn.
Schmiedebergs Arch. Pharmacol..

[25] Murray J. I., Whitfield M. L., Trinklein N. D., Myers R. M., Brown P. O., Botstein D. (2004). Diverse and Specific Gene Expression Responses
to Stresses in Cultured Human Cells. Mol. Biol.
Cell.

[26] Gray J. H., Owen R. P., Giacomini K. M. (2004). The concentrative
nucleoside transporter
family, SLC28. Eur. J. Physiol..

[27] Welin M., Nordlund P. (2010). Understanding specificity
in metabolic pathways—Structural
biology of human nucleotide metabolism. Biochem.
Biophys. Res. Commun..

[28] Maj M. C., Singh B., Gupta R. S. (2000). Structure-Activity
Studies on Mammalian
Adenosine Kinase. Biochem. Biophys. Res. Commun..

[29] Palella T. D., Andres C. M., Fox I. H. (1980). Human placental
adenosine kinase.
Kinetic mechanism and inhibition. J. Biol. Chem..

[30] Boison D. (2002). Neonatal hepatic steatosis
by disruption of the adenosine kinase
gene. Proc. Natl. Acad. Sci. U. S. A..

[31] Yegutkin G. G., Boison D. (2022). ATP and Adenosine Metabolism
in Cancer: Exploitation
for Therapeutic Gain. Pharmacol. Rev..

[32] Zavialov A. V., Yu X., Spillmann D., Lauvau G., Zavialov A. V. (2010). Structural Basis
for the Growth Factor Activity of Human Adenosine Deaminase ADA2. J. Biol. Chem..

[33] Johnston J. B. (2011). Mechanism
of Action of Pentostatin and Cladribine in Hairy Cell Leukemia. Leuk. Lymphoma.

[34] Wang D., Shalamberidze A., Arguello A. E., Purse B. W., Kleiner R. E. (2022). Live-Cell
RNA Imaging with Metabolically Incorporated Fluorescent Nucleosides. J. Am. Chem. Soc..

[35] Zhang Y., Kleiner R. E. (2019). A Metabolic Engineering Approach to Incorporate Modified
Pyrimidine Nucleosides into Cellular RNA. J.
Am. Chem. Soc..

[36] Wang D., Zhang Y., Kleiner R. E. (2020). Cell- and Polymerase-Selective Metabolic
Labeling of Cellular RNA with 2′-Azidocytidine. J. Am. Chem. Soc..

[37] Shimamoto Y. (2002). Sensitivity of Human Cancer Cells to the New Anticancer *Ribo* -nucleoside TAS–106 Is Correlated with Expression of Uridine-cytidine
Kinase 2. Jpn. J. Cancer Res..

[38] Sarkisjan D. (2016). The Cytidine Analog
Fluorocyclopentenylcytosine (RX-3117) Is Activated
by Uridine-Cytidine Kinase 2. PLoS One.

[39] Van
Kuilenburg A. B. P., Meinsma R. (2016). The pivotal role of uridine-cytidine
kinases in pyrimidine metabolism and activation of cytotoxic nucleoside
analogues in neuroblastoma. Biochim. Biophys.
Acta BBA - Mol. Basis Dis..

[40] Xu Z., Flensburg C., Bilardi R. A., Majewski I. J. (2023). Uridine–cytidine
kinase 2 potentiates the mutagenic influence of the antiviral β-d-N4-hydroxycytidine. Nucleic Acids Res..

[41] Lacroix E., Audas T. E. (2022). Keeping up with the condensates: The retention, gain,
and loss of nuclear membrane-less organelles. Front. Mol. Biosci..

[42] Boulon S., Westman B. J., Hutten S., Boisvert F.-M., Lamond A. I. (2010). The Nucleolus
under Stress. Mol. Cell.

[43] Yang K., Yang J., Yi J. (2018). Nucleolar
Stress: hallmarks, sensing
mechanism and diseases. Cell Stress.

[44] Lafontaine D. L. J., Riback J. A., Bascetin R., Brangwynne C. P. (2021). The nucleolus
as a multiphase liquid condensate. Nat. Rev.
Mol. Cell Biol..

[45] Lafita-Navarro M. C., Conacci-Sorrell M. (2023). Nucleolar stress: From development
to cancer. Semin. Cell Dev. Biol..

[46] Schoefl G. I. (1964). The effect
of actinomycin D on the fine structure of the nucleolus. J. Ultrastruct. Res..

[47] Reynolds R. C., Montgomery P. O., Hughes B. (1964). Nucleolar “Caps”
Produced
by Actinomycin D. Cancer Res..

[48] Potapova T. A., Unruh J. R., Conkright-Fincham J., Banks C. A., Florens L., Schneider D. A., Gerton J. L. (2023). Distinct states of nucleolar
stress induced by anticancer drugs. eLife.

[49] Dash S. (2023). rRNA transcription is integral to phase separation and maintenance
of nucleolar structure. PLOS Genet..

[50] Kang J., Brajanovski N., Chan K. T., Xuan J., Pearson R. B., Sanij E. (2021). Ribosomal proteins and human diseases: molecular mechanisms
and targeted therapy. Signal Transduct. Target.
Ther..

[51] Liu J., Xu Y., Stoleru D., Salic A. (2012). Imaging protein synthesis
in cells
and tissues with an alkyne analog of puromycin. Proc. Natl. Acad. Sci. U. S. A..

[52] Murakami E. (2011). Adenosine Deaminase-like
Protein 1 (ADAL1): Characterization and
Substrate Specificity in the Hydrolysis of N ^6^ - or O ^6^ -Substituted Purine or 2-Aminopurine Nucleoside Monophosphates. J. Med. Chem..

[53] Van
Rompay A. R., Norda A., Lindén K., Johansson M., Karlsson A. (2001). Phosphorylation of Uridine and Cytidine
Nucleoside Analogs by Two Human Uridine-Cytidine Kinases. Mol. Pharmacol..

[54] Preumont A., Snoussi K., Stroobant V., Collet J.-F., Van Schaftingen E. (2008). Molecular
Identification of Pseudouridine-metabolizing Enzymes. J. Biol. Chem..

[55] Chen M., Witte C.-P. (2020). A Kinase and a Glycosylase Catabolize Pseudouridine
in the Peroxisome to Prevent Toxic Pseudouridine Monophosphate Accumulation. Plant Cell.

[56] Chen J.-K. (2024). An RNA damage response network mediates the lethality of 5-FU in
colorectal cancer. Cell Rep. Med..

[57] Burger K. (2013). 4-thiouridine inhibits
rRNA synthesis and causes a nucleolar stress
response. RNA Biol..

[58] Lafita-Navarro M. C. (2020). Inhibition of the de
novo pyrimidine biosynthesis pathway limits
ribosomal RNA transcription causing nucleolar stress in glioblastoma
cells. PLOS Genet..

[59] Leppik M., Liiv A., Remme J. (2017). Random pseuoduridylation
in vivo
reveals critical region of Escherichia coli 23S rRNA for ribosome
assembly. Nucleic Acids Res..

[60] Delatte B. (2016). Transcriptome-wide distribution
and function of RNA hydroxymethylcytosine. Science.

